# Substantial Annual Distribution

**Published:** 1920-12-18

**Authors:** 


### KING EDWARD'S HOSPITAL FUND FOR LONDON.

### Substantial Annual Distribution.

A meeting of the President and General Council of King Edward's Hospital Fund for London, for the purpose of awarding grants to the hospitals, convalescent homes, and consumption sanatoria for the present year, was held on Tuesday morning at St. James's Palace, His Royal Highness the Prince of Wales being in the chair.

His Royal Highness The Prince of Wales moved the following resolution :—

"The President and General Council of King Edward's Hospital Fund for London desire to place on record their deep regret at the death of the Earl of Bessborough. He had done conspicuously able service to the Fund for many years as a member of the Council, Chairman of the Organising Committee of the Coronation Gift to His late Majesty King Edward, and member and Chairman of the Executive Committee, and the President and General Council greatly deplore his loss."

The resolution was put from the chair, the Council rising in their places, and carried unanimously.

Lord Revelstoke (the Hon. Treasurer) said that the amount received for general purposes for the period from January 1 to December 4, after deducting expenses, amounted to £156,880 5s. 5d. The original estimate for the whole year was £183,283.

Since the date on which this estimate was framed they had received the very generous offer of King George's Fund for Sailors to contribute an amount equal to the grant made to the "Dreadnought" Hospital—viz., £4,000. This last item would, therefore, have the effect of increasing the total of net estimated receipts to £187,283.

Sir William Collins, in making the annual statement on behalf of the League of Mercy, said that this had been a very difficult year, largely owing to the uncertainty prevailing as to the future relations between the State and the voluntary hospitals. He was, however, authorised to say that the League would be in a position to hand over £16,000 to King Edward's Fund this year. That would make a total, during the last twenty-one years, of £309,000 which the League of Mercy had handed over to the Fund. They had also made grants to the Extra-Metropolitan Hospitals to the amount of £37,393, making a grand total distributed by the League during the twenty-one years of £346,393.

The Prince of Wales, in moving the adoption of the report and awards (a list of which will be published in our next issue), said : My Lords, Ladies, and Gentlemen, I have the honour to read the following letter from His Majesty the King :—

"Buckingham Palace,
"December 14, 1920.

"Please assure the members of the Council of my unabated interest in the work of the Fund and of my appreciation of what it has done in helping to the best of its powers the London hospitals in this year of difficulty. The distribution of £450,000 out of our resources, in addition to the £250,000 given to the British Red Cross Society and the Order of St. John of Jerusalem through us, has been a great achievement.

"I am specially pleased to see that the collection organised by the teachers amongst the children of the London schools has been so conspicuously successful—over £22,000 has been raised, of which £10,000 is to be distributed by the Fund. This is indeed a substantial proof of the sympathetic interest of the rising generation and of their instructors in the work of our hospitals.

"As patron of King George's Fund for Sailors, I am gratified to know of its generous undertaking to provide this year's grant to the Seamen's 'Dreadnought' Hospital.

"It is my earnest hope that the annual subscriptions to the Fund which bears my father's name will be maintained. While we have to regret the loss of many old friends and supporters, it must be our endeavour to find fresh sources of help.

"I especially deplore the loss of the Chairman of the Executive Committee, the Earl of Bessborough, one of our most zealous and capable workers.

"The retirement of Mr. Griffiths, as Honorary Secretary, and Sir John Tweedy, as member and Chairman of the Distribution Committee, though well earned by long and valuable services, will be a serious loss to the Fund. I hope that both will retain their connection with the Council, and that the Executive Committee may still enjoy the benefit of Mr. Griffiths' vast knowledge and experience.

(Signed) "George, R.I.

"His Royal Highness, the President,
"King Edward's Hospital Fund for London."

It is now a whole year since I last presided at one of these meetings, and I am very pleased to be back again. There is one question of policy in regard to my position as President on which I should like to make a statement. It has been a great pleasure to me to be associated with several individual London hospitals. But I must own that now I have succeeded my father and grandfather in the office of President of the Fund I feel that my first duty is to promote its interests, and that this is the best way in which I can help the voluntary hospitals of London. It will, therefore, be my aim to devote myself, as far as lies in my power, to the carrying out of the objects for which the Fund was founded, and in so doing I am sure that I can rely on your aid.

The total amount distributed by the King's Fund this year is £700,000, the largest amount in any previous year being £230,000, in 1919. Of the £700,000, £250,000 was entrusted to the Fund by the Joint Committee of the British Red Cross Society and the Order of St. John of Jerusalem, for distribution on their behalf in aid of schemes of extension or improvement at hospitals able and willing to treat ex-Service men. This was distributed on July 23. Another £250,000 out of our own funds was distributed on July 5 in emergency grants to assist the maintenance of the hospitals in their pressing needs. The ordinary distribution made to-day is £200,000. The total amount given to maintenance this year is £414,000, as against £186,000 in 1919. The exceptional difficulties of the hospitals required this special effort. The King's Fund was founded to help to maintain the London voluntary hospitals. It is not responsible for their maintenance, but can only assist. I need hardly say that the emergency distribution in July did not itself provide a cure. It was intended to give time

**King Edward's Hospital Fund for London**—(*continued*)

for remedies to be devised. As you are aware, the Ministry of Health has announced that a Special Committee is about to be appointed to inquire into the position of the voluntary hospitals. The information in the possession of the Fund will be at the disposal of that Committee, and it is specially valuable as being independent evidence.

### Payments by Patients.

In the meantime the Executive Committee have been considering all suggestions that seemed likely to be at once useful, consistent with the maintenance of the voluntary system, and acceptable to the hospitals. When sending out the emergency grants in July the Fund asked all the hospitals to consider certain methods already adopted by some hospitals; for example, payment by patients able to pay and systematic collections from employers and employed.

The Executive Committee is now commending to the hospitals, through the British Hospitals Association, the suggestion that public authorities should pay the full cost of the hospital treatment of patients for whom those authorities have accepted responsibility. They have also appointed a sub-committee to consider the suggestion that contributions by donors might, subject to all necessary precautions and limitations, be exempted from income tax. Some people think this would be easy: others think it impossible. I feel sure that, from a sub-committee with Sir Walter Trower as its Chairman, the question will receive the thorough examination it deserves.

The Fund welcomes the decision of the National Relief Fund to distribute £700,000 amongst the hospitals to relieve deficits caused by the war. The emergency distribution of the King's Fund was given for current maintenance, and not to pay off war deficits. The National Relief Fund, like the Red Cross Society, takes into account hospitals outside London; and though the King's Fund in its active efforts is confined to London, we sympathise with the extra-Metropolitan hospitals in the difficulties which are common to all.

### The Statistical Report.

I have appointed a Committee, with Sir Arthur Stanley as Chairman, to consider in what way the Statistical Report, which had been reduced in size during the war, could best be enlarged, having regard to recent developments in hospital work.

I have already alluded to the sad death of Lord Bessborough. We have also to regret the deaths of Lord Murray of Elibank, Sir Henry Burdett, and Sir T. Vezey Strong, members of the Council, and of three visitors: Mr. Heathcote Long, Mr. Howard Morley, and Lord Glenconner.

The same, I think, applies to the retirement of Mr. John G. Griffiths, who has been an Honorary Secretary since 1912. He also carried through the revision of the uniform system of hospital accounts and statistics in 1906, and has placed his great skill and knowledge unreservedly at the disposal of the Fund. He will remain a member of the General Council and Executive Committee, and will, it is hoped, consent to serve on the Statistical Report Committee.

I am pleased to be able to announce that I propose to make the following new appointments to fill vacancies in the list of officers: Chairman of Executive Committee, Lord Stuart of Wortley; Chairman of Distribution Committee, Sir Cooper Perry; Honorary Secretary, Sir Alan Anderson.

On the motion of Viscount Burnham, seconded by the Bishop of London, and supported by Lord Stamfordham and Lord Somerleyton, it was resolved: "That it be an instruction to the Executive Committee to consider and report to the General Council what principles of policy should be recommended to His Majesty's Government for the preservation of the voluntary system of hospital management and control, and that a Special Meeting of the General Council be held to consider such report on the earliest day possible."

The Speaker of the House of Commons, in proposing a vote of thanks to the Prince of Wales for presiding, said that, whilst they felt that they owed a great debt to His Royal Highness for all the Empire work he had been carrying out abroad, they were rather jealous and rather anxious to see something of him at home. They were very gratified at the interest shown by His Royal Highness in the Fund, which was started by his grandfather. There were, indeed, very great problems facing all hospitals at the present moment, and they felt confident that the Council would be much fortified and strengthened by the interest His Royal Highness was taking, and that their work in support of the voluntary hospitals of London would be vastly benefited by his assistance.

The Bishop of London seconded the resolution, which was carried by acclamation.

The Prince of Wales, in acknowledging the vote of thanks, said he was very glad to be back home in England, when he would have the opportunity of attending the meetings of the Council more often than he had been able to do before.

The proceedings then terminated.

